# Dystrophin Deficiency Creates a Pro-Ferroptotic Environment in Diaphragm of mdx Mice That Is Modified by Diet and Glucocorticoid Treatment

**DOI:** 10.3390/biom16091347

**Published:** 2026-09-16

**Authors:** Morgan E. Vorwald, Melissa Roths, Rudy J. Valentine, Joshua T. Selsby

**Affiliations:** 1 Department of Animal Science, Iowa State University, 806 Stange Rd., Kildee Hall, Ames, IA 50010, USA; 2 Department of Physical Therapy and Kinesiology, University of Massachusetts Lowell, 114 Wilder Street, Suite 300, Lowell, MA 01854, USA

**Keywords:** ferroptosis, mdx, Duchenne muscular dystrophy, muscle, oxidative stress

## Abstract

Duchenne muscular dystrophy (DMD) is characterized by progressive muscle wasting with altered iron homeostasis and dysregulated lipid metabolism. Insulin resistance and obesity are common in DMD, but their impact on disease progression, particularly during glucocorticoid (GC) treatment, remains unclear. The role of ferroptosis, iron-dependent cell death, in DMD muscle pathology remains uncertain. We hypothesized that ferroptotic signaling would increase in mdx skeletal muscle, be further exacerbated by a high-fat, high-sucrose diet (HFHSD), and be attenuated by GC treatment. Male C57 and mdx mice were fed a control diet or HFHSD, with or without prednisolone, for 19 weeks before diaphragm western blot analysis. Dystrophic diaphragms had an increased abundance of transferrin (131%, *p* = 0.0006), transferrin receptor (72%, *p* = 0.0041), ferritin heavy chain (281%, *p* < 0.0001), acyl-CoA synthetase long-chain family member 4 (36%, *p* = 0.0080), and 5-lipoxygenase (101%, *p* = 0.0016) compared with C57. Despite these pro-ferroptotic changes, malondialdehyde and 4-hydroxynonenal were unchanged. Glutathione peroxidase 4 was robustly increased (800%, *p* < 0.0001), suggesting maintenance of redox balance and suppression of ferroptotic death. The HFHSD did not exacerbate ferroptotic signaling, whereas GC treatment partially offset disease-related changes in iron-handling and antioxidant proteins. These findings indicate that dystrophic diaphragms experience ferroptotic stress that is actively constrained, likely by compensatory GPX4 changes.

## 1. Introduction

Duchenne muscular dystrophy (DMD) is a fatal, progressive muscle wasting disease caused by a dystrophin protein deficiency that leads to sarcolemma fragility and increased susceptibility of contraction-induced injury [1,2]. The subsequent membrane lesions challenge homeostatic control of Ca^2+^ [3] and Fe^2+^/Fe^3+^ [4,5], among other small ions and molecules, which may promote oxidative stress and further contribute to membrane damage and muscle degeneration [6,7]. Loss of iron homeostatic control can trigger ferroptosis, an iron-dependent, caspase-independent form of cell death that is initiated by intracellular ferrous iron and lipid peroxide accumulation [8,9]. Under healthy conditions, glutathione peroxidase 4 (GPX4) inhibits reactive oxygen species (ROS)-induced lipid peroxidation, especially catalyzed by lipoxygenases, thereby preventing ferroptosis [10]. Impaired activity or inadequate abundance of GPX4 promotes accumulation of oxidatively modified polyunsaturated fatty acids (PUFAs) [11]. These oxidized PUFAs undergo fragmentation, producing lipid peroxides and secondary reactive aldehydes, including malondialdehyde (MDA) and 4-hydroxynonenal (4HNE) [12,13,14,15], which promote cell death by permeabilizing lipid membranes [9,16]. Consequently, lipid peroxides can accelerate further oxidation of membrane phospholipids, damage proteins and nucleic acids, and ultimately, contribute to cellular dysfunction and death [16,17].

In humans, DMD is frequently complicated by metabolic dysfunction, including obesity and insulin resistance (IR) [18,19,20,21,22], and is commonly treated by long-term use of glucocorticoids (GC) [23,24]. Ferroptosis can be increased by a high fat diet (HFD) due, at least in part, to increased dietary PUFAs, which provide additional substrates for lipid peroxidation [25,26]. These changes are frequently coupled with diminished glutathione synthesis, GPX4 activity [26], and antioxidant defenses [27] that create conditions that collectively favor ferroptosis. Likewise, GC treatment alters lipid composition of membranes, leading to increased free fatty acids [28] that can increase lipid peroxidation substrates [29]. In addition, GCs can limit antioxidant defenses via inhibition of glutathione synthesis and GPX4 activity [30]. GCs have also been implicated in disruption of iron homeostasis, which may further amplify ROS production via Fenton chemistry, a reaction that uses ferrous iron and hydrogen peroxide to produce hydroxyl radicals [31]. Despite these risks and known side-effects, GCs decrease disease severity caused by dystrophin deficiency and consistently improve muscle strength, prolong ambulation, and suppress inflammation [32,33], making their impact on ferroptosis difficult to predict in dystrophic muscle. Nevertheless, a HFD and/or GCs may contribute to a metabolic and redox environment that promotes accumulation of lipid peroxides and could enhance susceptibility to ferroptotic cell death.

Although ferroptosis has been proposed to contribute to disease progression in DMD [4,34], whether ferroptosis occurs in dystrophic muscle, and the extent to which a high-fat, high-sucrose diet (HFHSD) and GC treatment further modify ferroptosis within the context of dystrophic muscle, remain unknown. Therefore, the purpose of this study was to assess ferroptotic signaling in muscle from mdx mice and evaluate the impact of a HFHSD and/or GC on ferroptotic signaling in dystrophin-deficient muscle. We hypothesized that markers of ferroptotic signaling would increase in skeletal muscle from mdx mice, would be further exacerbated by a HFHSD and, as GC ultimately provides an overall therapeutic effect, would be attenuated by GC treatment.

## 2. Materials and Methods

### 2.1. Animal Treatments

All experimental procedures were reviewed and approved by the Institutional Animal Care and Use Committee at Iowa State University, Ames, IA, USA (IACUC-22-162; approved 4 August 2022). A detailed study design and animal treatment have been previously described [35]. Briefly, four-week-old mdx (C57BL/10ScSn-Dmd^mdx^/J) were procured from Jackson Laboratories and at 6 w were assigned to one of four treatments: control diet (3.82 kcal/g; D12450K Research Diets, Inc.—New Brunswick, NJ, USA; composed of 20% protein, 10% fat, and 70% carbohydrates), control diet with prednisolone (Pred; 10 mg/L in water), a HFHSD (4.7 kcal/g; D12451 Research Diets, Inc.; composed of 20% protein, 45% fat, 35% carbohydrates, and sucrose constituting 20% of diet by weight), and HFHSD with Pred. Male C57 (C57BL/10ScSnJ) mice on a control diet without Pred served as healthy controls. Assigned diets and water were given ad libitum. At 25 weeks of age, mice were sedated to a surgical plane of anesthesia using a ketamine/xylazine cocktail and costal diaphragm was collected. All groups were initially assigned *n* = 10; however, due to attrition that occurred during this long-term investigation, final group sizes were C57 CD (*n* = 10), mdx CD (*n* = 7), mdx CD Pred (*n* = 9), mdx HFHSD (*n* = 9), and mdx HFHSD Pred (*n* = 10). The groups are defined as follows: C57-CD-no Pred (CCN), mdx-CD-no Pred (MCN), mdx-CD-Pred (MCP), mdx-HFHSD-no Pred (MHN), and mdx-HFHSD-Pred (MHP).

### 2.2. Western Blot

A portion of the hemi-diaphragm was collected, snap-frozen in liquid nitrogen, and stored at −80 °C. Protein extraction and Western blotting were performed as previously described [35,36]. Briefly, whole muscle protein was extracted from powdered diaphragm tissue using protein extraction buffer (10 mM Sodium Phosphate buffer, pH 7.0, 2% SDS) with a protease and phosphatase inhibitor (Halt protease and phosphatase inhibitor cocktail, Thermo Fisher Scientific, Waltham, MA, USA). Samples were homogenized on ice, centrifuged, and the supernatant was collected. Protein concentration was measured using Pierce BCA Protein Assay Kit (Thermo Fisher Scientific, 23225) according to the manufacturer’s instructions.

Samples were loaded onto 4–20% Criterion TGX precast midi protein gels (#5671095, Bio-Rad, Hercules, CA, USA), separated by electrophoresis, and transferred onto nitrocellulose membranes (#1620112, Bio-Rad). Equal protein loading was verified via quantification of Ponceau S staining using an Azure Biosystems c600 imaging system (Azure Biosystems, Dublin, CA, USA). Membranes were washed in TBST (50 mmol/L Tris·HCl, 150 mmol/L NaCl, 0.1% Tween 20, pH 7.4), blocked in 5% nonfat dehydrated milk dissolved in TBST, washed again with TBST, and incubated with primary antibodies overnight at 4 °C (Table 1). Membranes were washed in TBST, incubated in species-specific secondary antibody at room temperature, washed again, and then incubated with enhanced chemiluminescence (Bio-Rad). Protein bands were imaged using Azure Biosystems c600 imaging system and quantification of bands was done using AzureSpot Software (version 2.2.167) with automated band detection. Data were normalized to CCN.

### 2.3. Statistics

Normality was confirmed before statistical analysis using the Shapiro–Wilk test. Non-normally distributed data were log10-transformed before analysis and normality confirmed. Analysis was completed using PROC MIXED procedure in SAS version 9.4 (SAS Institute Inc., Cary, NC, USA) with the model including diet, treatment, and the interaction between diet and treatment. Preplanned contrasts were used to evaluate the effects of diet and/or Pred within disease (e.g., mdx-CD vs. mdx-HFHSD) and the effects of disease within a treatment (i.e., C57-CD vs. mdx-CD). Outliers were objectively identified using the ROUT method (Q = 1%) in GraphPad Prism software (version 8.2.0). Data are reported as mean ± standard deviation (SD). Statistical significance was defined as *p* < 0.05.

## 3. Results

We have previously established that feeding a HFHSD induced hyperglycemia, glucose intolerance, and insulin resistance in mdx mice (Table 2) [35]. Further, in these diaphragms, a HFHSD increased adiposity, decreased fibrotic accumulation, and increased fatigue suggestive of additional metabolic stress in dystrophic pathology. Glucocorticoid treatment improved muscle function, reduced fibrosis, inflammatory signaling, and mitochondrial dysregulation but increased fatty infiltration. Additionally, the HFHSD blunted some glucocorticoid-mediated benefits.

**Figure 1:**
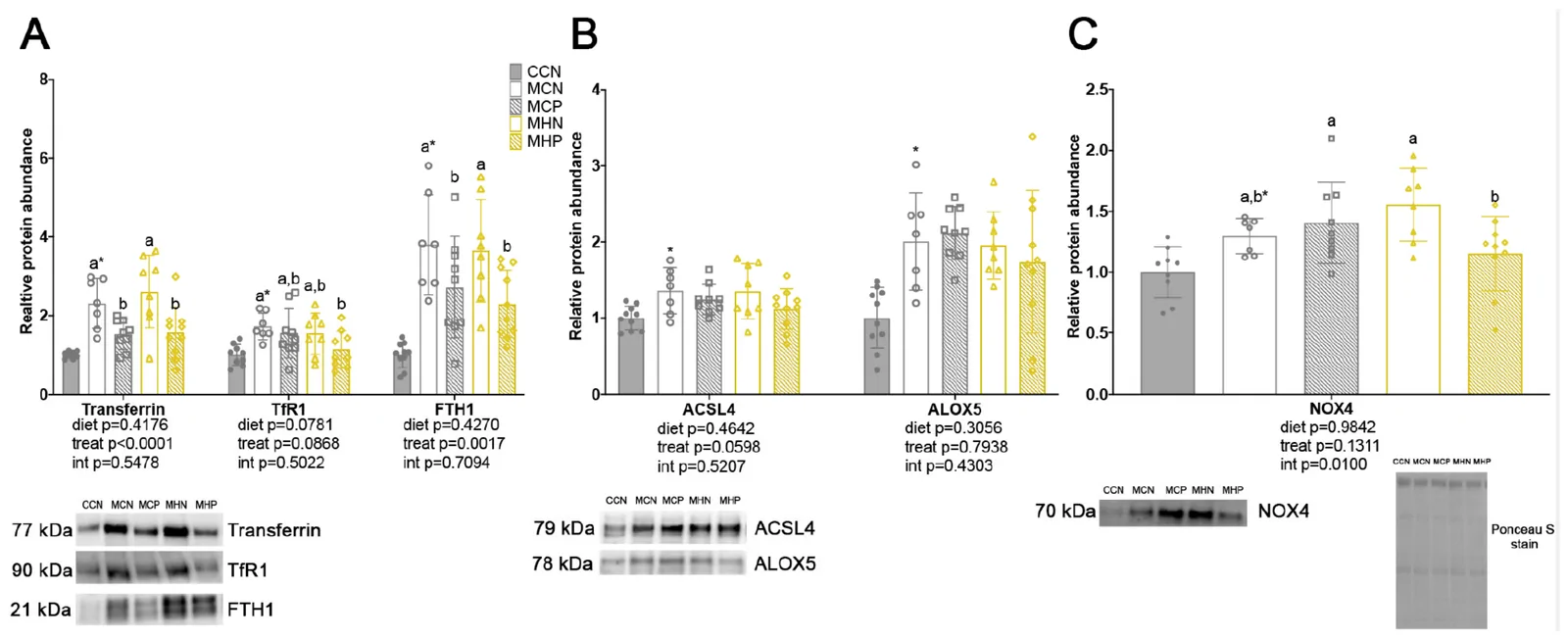
Iron-handling proteins and pro-ferroptotic markers were increased in mdx diaphragms. (**A**) Transferrin, transferrin receptor (TfR1), and ferritin heavy chain (FTH1) were measured using western blot. One outlier was removed from the CCN transferrin group and one outlier was removed from the MHP TfR1 group. Quantification and representative blots for (**B**) pro-ferroptotic markers Acyl-coenzyme A synthetase long-chain family member 4 (ACSL4) and 5-lipoxygenase (ALOX5) and (**C**) NADPH oxidase 4 (NOX4). One outlier was removed from the CCN NOX4 group. Final group sizes were C57 CD (*n* = 10), mdx CD (*n* = 7), mdx CD-Pred (*n* = 9), mdx-HFHSD (*n* = 9), and mdx HFHSD-Pred (*n* = 10). Groups are indicated by genotype: C57 (C) or mdx (M); diets are designated as control diet (C) or HFHSD (H); and treatment is designated as no Pred (N) or Pred (P). Significance is established at *p* < 0.05. Main effects of diet and treatment [Pred] and the interaction are indicated below each graph. Different lowercase letters indicate differences between mdx groups (*p* < 0.05). * indicates differences between CCN and MCN groups (*p* < 0.05). Data are presented as mean ± SD. The original western blot images can be found in the Supplementary Materials.

Iron-handling proteins including transferrin, transferrin receptor (TfR1), and ferritin heavy chain (FTH1) regulate the uptake and storage of iron within the cell [37,38]. Transferrin increased 131% in MCN compared to CCN (*p* = 0.0002; Figure 1A) and, within mdx mice, decreased as a main effect of Pred (*p* < 0.0001). Furthermore, transferrin decreased 35% in MCP compared to MCN (*p* = 0.0128), and MHP decreased 40% compared to MHN (*p* = 0.0008). TfR1 increased 72% in MCN compared to CCN (*p* = 0.0041). The combination of the HFHSD and GC decreased TfR1 abundance 34% in MHP compared to MCN (0.0178). FTH1 increased 281% in MCN compared to CCN (*p* < 0.0001) and decreased as a main effect of Pred (*p* = 0.0017). Further, FTH1 decreased 29% in MCP compared to MCN (*p* = 0.0459) and decreased 37% in MHP compared to MHN (*p* = 0.0093).

Acyl-coenzyme A synthetase long-chain family member 4 (ACSL4) and 5-lipoxygenase (ALOX5) contribute to ferroptosis by promoting the incorporation and oxidation of PUFAs within membrane phospholipids and increasing the susceptibility of membrane lipids to peroxidation [39,40]. ACSL4 increased 36% (*p* = 0.0080; Figure 1B) and ALOX5 increased 101% (*p* = 0.0016) in MCN compared to CCN, and both proteins were similar within mdx groups. NADPH oxidase 4 (NOX4) promotes ferroptosis by increasing ROS production [41]. NOX4 increased 30% in MCN compared to CCN (*p* = 0.0372; Figure 1C) and there was a significant interaction of diet*treatment (*p* = 0.0100), with MHP decreased 20% compared to MHN (*p* = 0.0036).

**Figure 2:**
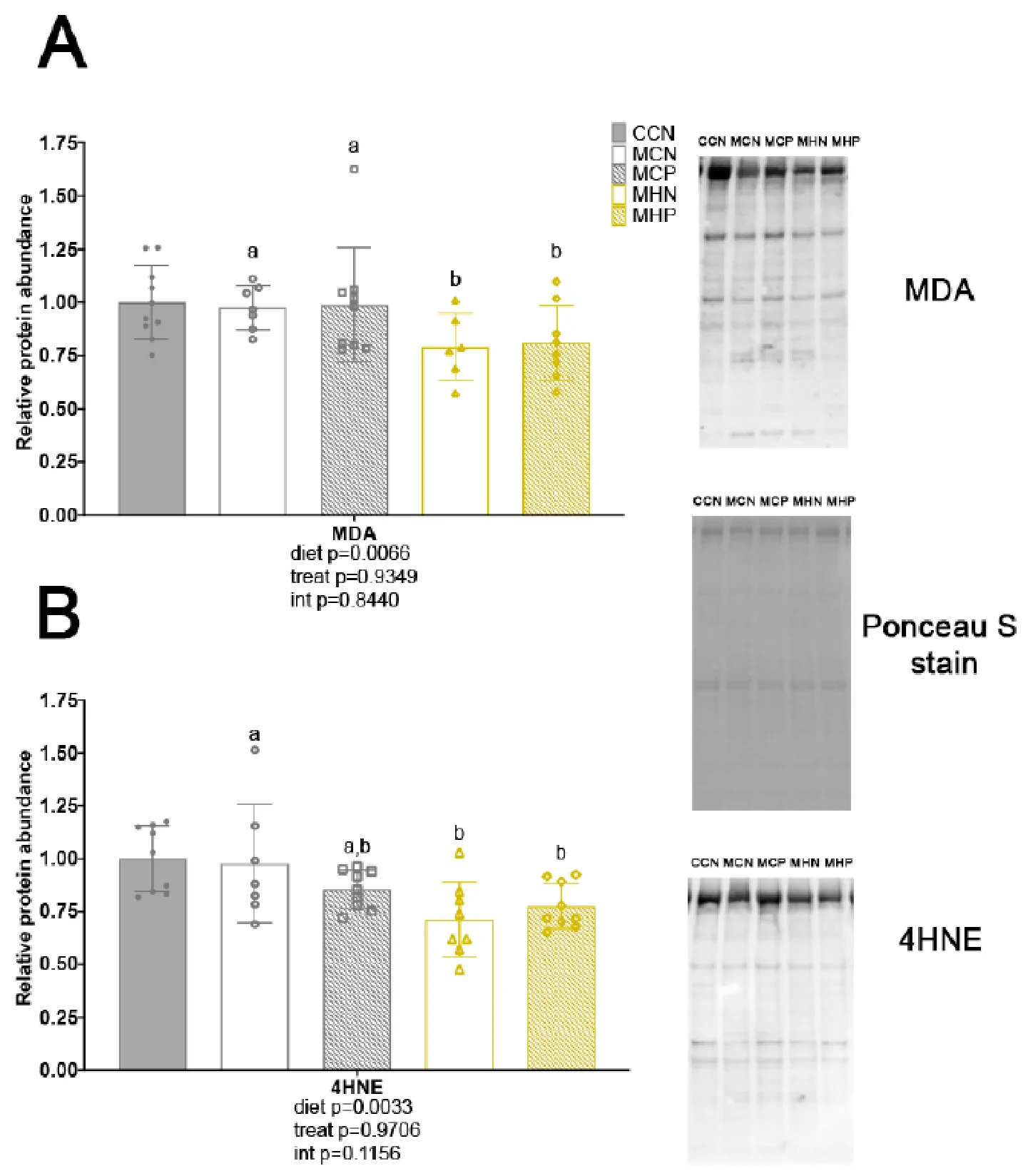
Markers of lipid peroxidation were similar between mdx and C57 diaphragms. Quantification and representative blots for (**A**) malondialdehyde (MDA) and (**B**) 4-hydroxynoneal (4HNE). One outlier was removed from the MHN MDA group. Final group sizes were C57 CD (*n* = 10), mdx CD (*n* = 7), mdx CD-Pred (*n* = 9), mdx-HFHSD (*n* = 9), and mdx HFHSD-Pred (*n* = 10). Groups are indicated by genotype: C57 (C) or mdx (M); diets are designated as control diet (C) or HFHSD (H); and treatment is designated as no Pred (N) or Pred (P). Significance is established at *p* < 0.05. The main effects of diet and treatment [Pred] and the interaction are indicated below each graph. Different lowercase letters indicate differences between mdx groups (*p* < 0.05). Data are presented as mean ± SD. The original western blot images can be found in the Supplementary Materials.

Accumulation of lipid peroxides, driven by an imbalance of free radical production and antioxidant defenses, can result in oxidative stress, an essential component of ferroptosis [42]. Relative protein abundance of malondialdehyde (MDA; Figure 2A) and 4-hydroxynoneal (4HNE; Figure 2B) were similar between healthy (CCN) and diseased (MCN) diaphragms, and, surprisingly within mdx mice, both MDA and 4HNE decreased as a main effect of diet (*p* = 0.0066 and *p* = 0.0033, respectively). Further, MDA decreased 13% in MHN compared to MCN (*p* = 0.0480) and 12% in MHP compared to MCN (*p* = 0.0484). 4HNE decreased 27% in MHN and 21% in MHP compared to MCN (*p* = 0.0027 and *p* = 0.0336, respectively).

**Figure 3:**
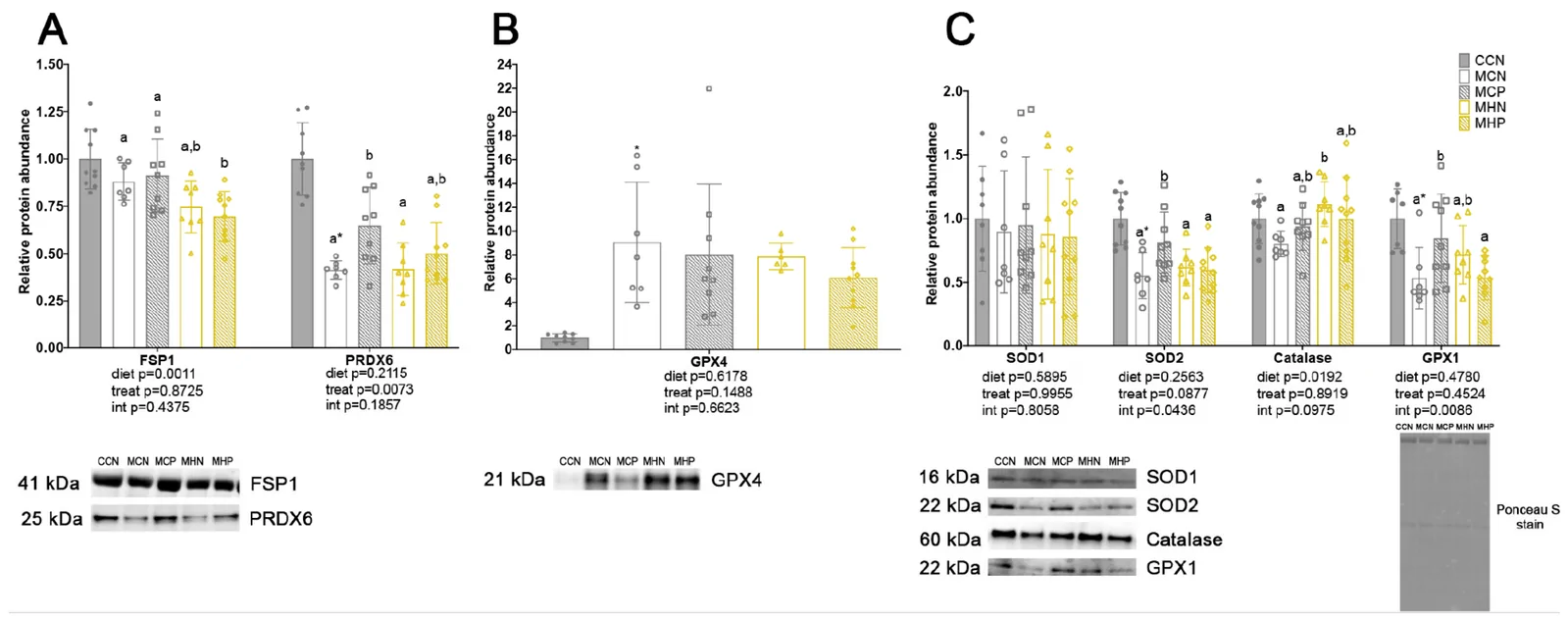
Glutathione peroxidase 4 (GPX4) increased by disease while other antioxidant markers decreased by disease. (**A**) Ferroptosis suppressing protein (FSP1) and peroxiredoxin 6 (PRDX6) were measured. Quantification and representative blots for (**B**) GPX4 and (**C**) superoxide dismutase 1 (SOD1), superoxide dismutase 2 (SOD2), catalase, and glutathione peroxidase 1 (GPX1). Two outliers were removed from the MHN GPX4 group. Final group sizes were C57 CD (*n* = 10), mdx CD (*n* = 7), mdx CD-Pred (*n* = 9), mdx-HFHSD (*n* = 9), and mdx HFHSD-Pred (*n* = 10). Groups are indicated by genotype: C57 (C) or mdx (M); diets are designated as control diet (C) or HFHSD (H); and treatment is designated as no Pred (N) or Pred (P). Significance is established at *p* < 0.05. The main effects of diet and treatment [Pred] and the interaction are indicated below each graph. Different lowercase letters indicate differences between mdx groups (*p* < 0.05). * indicates differences between CCN and MCN groups (*p* < 0.05). Data are presented as mean ± SD. The original western blot images can be found in the Supplementary Materials.

Peroxiredoxin 6 (PRDX6), glutathione peroxidase 4 (GPX4), and ferroptosis-suppressing proteins like ferroptosis suppressor protein (FSP1) protect against ferroptotic death [10,12,43]. FSP1 was similar between MCN and CCN and decreased as a main effect of diet in dystrophic diaphragms (*p* = 0.0017; Figure 3A). Additionally, FSP1 decreased 24% in MHP compared to MCP (*p* = 0.0033). PRDX6 decreased 59% in MCN compared to CCN (*p* < 0.0001) and increased as a main effect of Pred (*p* = 0.0073), with MCP increased 57% compared to MCN (*p* = 0.0066). GPX4 increased 807% in dystrophic diaphragms compared to healthy diaphragms (*p* < 0.0001; Figure 3B) and was similar between all mdx groups.

To better understand the maintained redox balance in dystrophic muscle, we measured the relative protein abundance of additional antioxidants, including superoxide dismutase 1 (SOD1), superoxide dismutase 2 (SOD2), catalase, and glutathione peroxidase 1 (GPX1). Superoxide dismutase 1 (SOD1) was similar between all groups (Figure 3C). Superoxide dismutase 2 (SOD2) decreased 45% in MCN compared to CCN (*p* < 0.0001) and there was a significant interaction of diet*treatment (*p* = 0.0436), such that GC-mediated restoration of SOD2 was blocked by the HFHSD. Additionally, SOD2 increased 47% in MCP compared to MCN (*p* = 0.0120) and 24% compared to MHN (*p* = 0.0453), but the addition of the HFHSD blocked this GC-mediated increase of SOD2, such that MHP was 27% lower than MCP (*p* = 0.0194). Catalase was similar between CCN and MCN and, in mdx mice, increased as a main effect of diet (*p* = 0.0192). Catalase increased 39% in MHN compared to MCN (*p* = 0.0086). GPX1 decreased 55% in MCN compared to CCN (*p* = 0.0015) and there was a significant interaction of diet*treatment (*p* = 0.0086), such that GC-mediated elevations in GPX1 were blocked by the HFHSD. Further, within mdx groups, GPX1 increased 89% in MCP compared to MCN (*p* = 0.0189) and MHP decreased 31% compared to MCP (*p* = 0.0136).

## 4. Discussion

In DMD, iron dysregulation and increased ROS production may promote lipid peroxidation through Fenton chemistry, while simultaneous reductions in antioxidant capacity may further increase susceptibility to ferroptotic cell death reaction [4,7,13,34,44,45,46,47]. DMD is frequently complicated by obesity and IR, which further undermine metabolic health and may contribute to ferroptosis [18,19,20,21,22]. Similarly, GCs are a commonly used treatment in DMD [23,24], although their impact on ferroptosis in DMD is unknown. The purpose of this study was to determine if ferroptotic signaling in dystrophic skeletal muscle increased and to evaluate the impact of IR and/or GC on ferroptotic signaling. We hypothesized that ferroptosis would be increased in dystrophic muscle, that it would be further increased by a HFHSD, and attenuated by GC. We discovered that dystrophin deficiency created a pro-ferroptotic environment; however, this was countered by a dramatic elevation in GPX4, which appears sufficient to maintain redox balance, and thus stymie this culminating step in ferroptosis. Surprisingly, a HFHSD did not exacerbate lipid peroxidation, and even decreased lipid peroxidation levels compared to controls, counter to our hypothesis. Glucocorticoids decreased iron handling proteins and increased antioxidant enzymes, supporting their protective role against ferroptosis, consistent with our hypothesis. Notably, the HFHSD blocked some GC-mediated benefits, consistent with our recent discoveries [35].

Dystrophin deficiency increased iron dysregulation, which, alongside elevations in iron-trafficking proteins and pro-ferroptotic enzymes, created a ferroptotic-primed intracellular environment in mdx diaphragms. Increased ferric iron, which has been found in diaphragm, heart, and limb muscle of mdx mice [4,34], may expand the pool of free iron capable of catalyzing the Fenton reaction and promoting lipid peroxidation. Importantly, iron is essential for normal muscle physiology [48,49], whereas increased abundance of iron handling-proteins may reflect pathological iron accumulation as occurs in dystrophin-deficient muscle [4,5]. Concurrently, elevated ACSL4, which has been previously reported in mdx skeletal muscle [50], supports the incorporation of PUFAs, particularly arachidonic acid, into membrane phospholipids, which may increase membrane susceptibility to oxidative damage due to PUFA accumulation, and creates an environment conducive to ferroptotic cell death [39,51,52]. In this context, ALOXs play a critical role linking PUFA availability to ferroptotic signaling by catalyzing the oxygenation of free and membrane-bound PUFAs that can propagate lipid peroxidation and contribute to membrane damage [11,40]. The interaction between ACSL4 and ALOX5 in dystrophic diaphragm creates a highly permissive environment for ferroptosis as their simultaneous elevation suggests increased substrate availability and enzymatic capacity to generate lipid peroxides [52].

Despite an apparent pro-ferroptotic environment, lipid peroxidation was not increased by dystrophin deficiency. This was surprising as dystrophin deficiency has been associated with increased oxidative stress, likely originating from mitochondrial dysfunction, NADPH oxidase activity, and chronic inflammation [53,54]. Moreover, in the diaphragms considered herein, reductions in antioxidant enzymes and simultaneous elevations in pro-oxidants favor oxidative damage. While unexpected given this larger context, it is reasonable to suggest the substantial increase of GPX4, a selenoprotein that is uniquely capable of directly reducing lipid hydroperoxides, is working to prevent lipid peroxide accumulation and maintain redox balance [10,55,56]. In contrast, previous studies have reported similar GPX4 abundance in dystrophic hearts compared to healthy hearts [34], and changes in GPX4 transcript in dystrophic muscle are equivocal as decreased and increased transcript has been previously reported [50], raising the possibility that GPX4 is altered by disease progression or is tissue-specific. The robust elevation of GPX4 in dystrophic diaphragms may represent a compensatory response to the pro-ferroptotic environment that prevents accumulation of oxidized lipids.

Glucocorticoid treatment decreased iron-handling proteins, indicating improved iron homeostasis, and further increased antioxidant enzymes such as PRDX6, SOD2, and GPX1 in CD-fed mice, suggesting enhanced capacity to neutralize ROS. This upregulation indicates that GCs, besides being anti-inflammatory, may reinforce redox balance, directly or indirectly, and complement GPX4-mediated reduction of lipid peroxides [57]. The expanded antioxidant capacity with GC treatment may be a compensatory mechanism to increased mitochondrial stress and ROS production associated with chronic glucocorticoid exposure [58]. PRDX6 may influence susceptibility to ferroptosis as it has both peroxidase and phospholipase activities [59], which have been associated with decreased oxidized phospholipids and repair of damaged phospholipids [60,61]. These functions support removal or repair of peroxidized phospholipids.

We anticipated that the additional metabolic stress imposed by the HFHSD would promote ferroptotic signaling by increasing availability of lipid substrates for peroxidation. However, most ferroptotic markers were resistant to diet-induced changes, and lipid peroxidation was unexpectedly decreased, raising the unanticipated possibility of a reductive stress. One explanation is that the HFHSD remodeled the lipid membrane composition with higher amounts of monounsaturated fatty acids (MUFAs) that are resistant to lipid peroxidation in comparison to PUFAs [62,63].

An important outcome of this work is that the HFHSD blunted GC-mediated changes to a variety of antioxidant proteins. This is in good agreement with our previous findings demonstrating a HFHSD interaction with GC resulted in enhanced hyperglycemia, greater impairments to glucose and insulin tolerance, blocking of restored mitochondrial abundance, and attenuation of functional correction [35]. Ostensibly, the HFHSD interferes with essential signaling networks necessary for GC efficacy. These findings suggest that while GCs can contribute to maintenance of lipid peroxidation, this is context-dependent [64,65] and may be compromised with additional metabolic stress from diet, further highlighting an important interaction of diet and therapeutic efficacy in dystrophic skeletal muscle.

There are several limitations to consider when interpreting the data in this study. First, the mdx mouse develops a relatively mild phenotype compared to individuals with DMD; however, the diaphragm of mdx mice at this age more closely recapitulates human DMD pathology than limb muscle [66]. Indeed, by 6 mo of age the diaphragm has dramatically increased fibrosis, evidence of muscle degeneration and regeneration, immune cell infiltration, and impaired muscle function, among other changes. Second, our findings identify alterations in proteins associated with iron handling, although iron was not directly quantified, nor did we measure all proteins and processes involved with iron-handling. Thus, our findings should be interpreted as evidence of alterations of ferroptosis-related signaling rather than definitive evidence of increased iron, per se. Nevertheless, previous evidence of iron dysregulation in DMD [4,5,34], along with coordinated alterations in iron-handling and ferroptotic signaling proteins reported herein, provide strong rationale to support further investigation of iron associated proteins and ferroptosis. Finally, although there was marked upregulation of GPX4, suggesting an enhanced antioxidant response that may limit ferroptotic cell death, the present study does not mechanistically establish that GPX4 provided functional protection against ferroptotic cell death, although it does raise this intriguing possibility, and we suggest this as a focus for future mechanistic investigation. Collectively, these limitations highlight the need for future studies directly measuring iron availability, lipid peroxidation, and ferroptotic cell death to determine the extent to which ferroptosis represents a clinically relevant cell death mechanism that contributes to DMD pathology.

## 5. Conclusions

These data suggest that dystrophin deficiency drives pro-ferroptotic signaling; however, ferroptotic death appears to be constrained by substantial upregulation of GPX4 and subsequent maintenance of redox balance, at least at this stage of disease. Nevertheless, these muscles may be on the precipice of ferroptosis, as more advanced disease may compromise GPX4 and increase the vulnerability to ferroptotic cell death. The addition of a HFHSD did not exacerbate ferroptosis and may even promote resistance to oxidative stress, whereas GC treatment enhanced antioxidant defenses. Notably, when in combination, the HFHSD may limit some aspects of GC-mediated disease mitigation. Future studies should directly assess iron availability and ferroptotic cell death throughout disease progression to determine whether the pro-ferroptotic signaling identified herein progresses to ferroptosis. Studies examining iron chelation strategies may provide insight into the therapeutic potential of targeting iron dysregulation in DMD. More broadly, these findings reinforce the importance of consideration of metabolic health as a modifier of disease progression and therapeutic response, particularly given the potential long-term metabolic consequences associated with GC treatment.

## Figures and Tables

**Table 1:** Antibodies and dilutions used for Western blotting diaphragm whole muscle extract.

Antibody	Company	Product Details	Host	Primary	Secondary
4HNE	Sigma ^1^	AB5605	Goat	1:1000 TBST ^6^	1:2000 TBST
ACSL4	Proteintech ^2^	22401	Rabbit	1:1000 TBST	1:1000 TBST
ALOX5	Proteintech	10021	Rabbit	1:1000 TBST	1:1000 TBST
Catalase	Cell Signaling ^3^	14097	Rabbit	1:1000 TBST	1:1000 TBST
CD71/TfR1	Proteintech	10084	Rabbit	1:500 TBST	1:500 TBST
FTH1	Santa Cruz ^4^	376594	Mouse	1:1000 TBST	1:1000 TBST
FSP1	Proteintech	20886	Rabbit	1:1000 TBST	1:1000 TBST
GPX1	Cell Signaling	3286	Rabbit	1:2000 TBST	1:2000 TBST
GPX4	Santa Cruz	166437	Mouse	1:1000 TBST	1:1000 TBST
MDA	Abcam ^5^	ab27642	Rabbit	1:1000 TBST	1:1000 TBST
NOX4	Santa Cruz	518092	Mouse	1:1000 TBST	1:1000 TBST
SOD1	Abcam	ab51254	Rabbit	1:500 TBST	1:500 TBST
SOD2	Abcam	ab13533	Rabbit	1:500 TBST	1:500 TBST
Transferrin	Proteintech	17435	Rabbit	1:500 TBST	1:500 TBST

^1^ Sigma—Burlington, MA, USA; ^2^ Proteintech—Rosemont, IL, USA; ^3^ Cell Signaling—Danvers, MA, USA; ^4^ Santa Cruz—Santa Cruz, CA, USA; ^5^ Abcam—Waltham, MA, USA; ^6^ TBST—Tris Buffered Saline with Tween 20.

**Table 2:** Biometric data modified from Vorwald et al., 2026 [35].

Characteristic	CCN	MCN	MCP	MHN	MHP
Final body weight (g)	31.26 ± 1.80	36.53 ± 1.94 ^a^*	28.44 ± 2.37 ^b^	38.30 ± 1.67 ^a^	36.68 ± 3.75 ^a^
Body fat (%)	1.98 ± 3.20	0.26 ± 0.84 ^a^	−0.96 ± 1.31 ^a^	3.64 ± 2.46 ^b^	3.79 ± 1.99 ^b^
Fasting glucose (12 h; mg/dL)	87.80 ± 16.19	76.71 ± 12.33 ^a^	80.78 ± 19.23 ^a^	125.88 ± 14.71 ^b^	145.90 ± 16.63 ^b^
Area under the curve GTT	611.80 ± 91.52	704.67 ± 92.24 ^a^	803.50 ± 206.50 ^a^	1197.56 ± 335.82 ^b^	1755.20 ± 208.17 ^c^
Area under the curve ITT	172.85 ± 43.44	169.29 ± 42.25 ^a^	155.00 ± 50.12 ^a^	257.29 ± 43.88 ^b^	304.11 ± 40.07 ^c^
Diaphragm fatty infiltration (%)	6.42 ± 5.30	5.88 ± 6.17 ^a^	12.87 ± 3.96 ^b^	15.02 ± 6.42 ^b^	12.09 ± 6.13 ^b^

Data are reported as mean ± SD. Groups are indicated by genotype: C57 (C) or mdx (M); diet are designated as control diet (C) or HFHSD (H); and treatment is designated as no Pred (N) or Pred (P). Different lowercase letters indicate differences between mdx groups (*p* < 0.05). * indicates differences between MCN and CCN (*p* < 0.05).

## Data Availability

The original contributions presented in this study are included in the article. Further inquiries can be directed to the corresponding author.

## Supplementary Materials

The following supporting information can be downloaded at: https://www.mdpi.com/article/10.3390/biom16091347/s1. The original western blot images can be found in this section.
