# Supplementary material for: Dystrophin Deficiency Creates a Pro-Ferroptotic Environment in Diaphragm of mdx Mice That Is Modified by Diet and Glucocorticoid Treatment
Source: Biomolecules. 2026 Sep 16;16(9):1347. doi: 10.3390/biom16091347 (PMC13604109; doi:10.3390/biom16091347)

Full Western blot images

"Dystrophin deficiency creates a pro-ferroptotic environment that is modified by diet and glucocorticoid treatment"

Transferrin

mem53

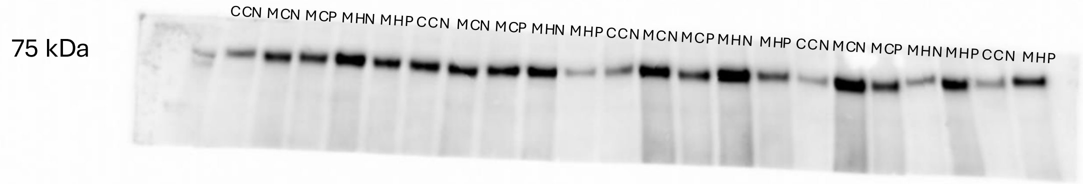

mem54

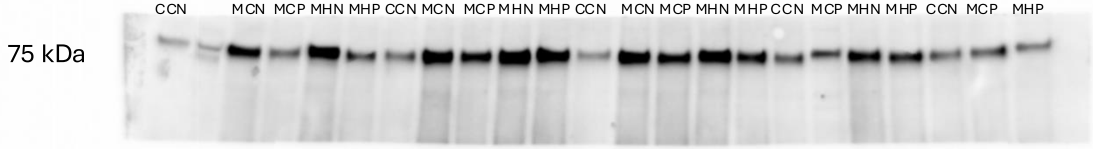

TfR1 (CD71)

mem55

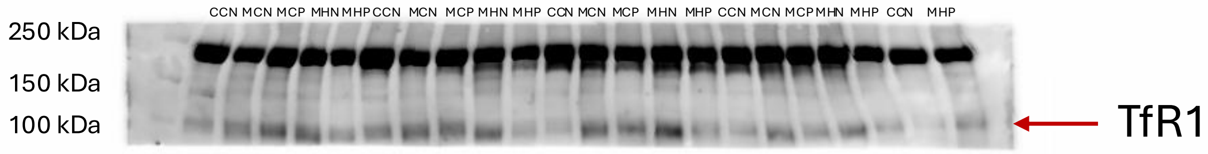

mem56

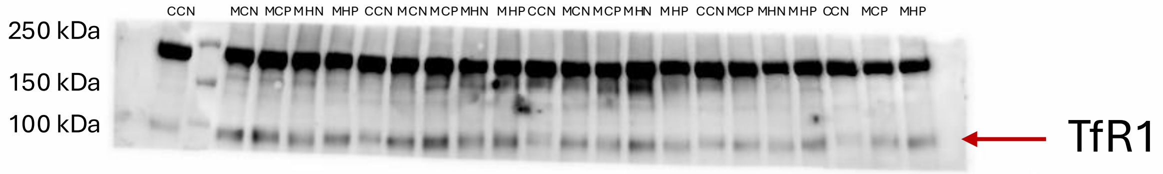

FTH1

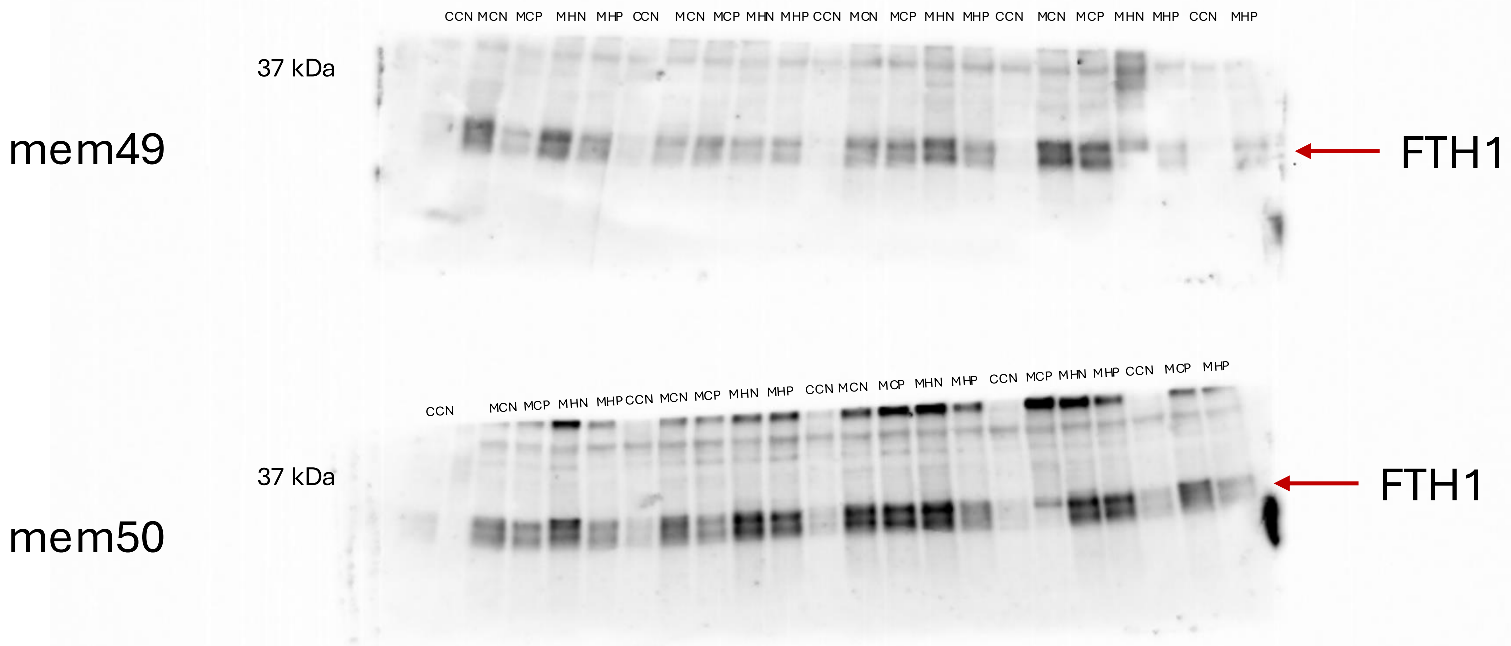
CCN MCN MCP MHN MHP CCN MCN MCP MHN MHP CCN MCN MCP MHN MHP CCN MCN MCP MHN MHP CCN MHP
37 kDa
mem49
FTH1
CCN MCN MCP MHN MHP CCN MCN MCP MHN MHP CCN MCN MCP MHN MHP CCN MCP MHN MHP CCN MCP MHP
37 kDa
mem50
FTH1

ACSL4

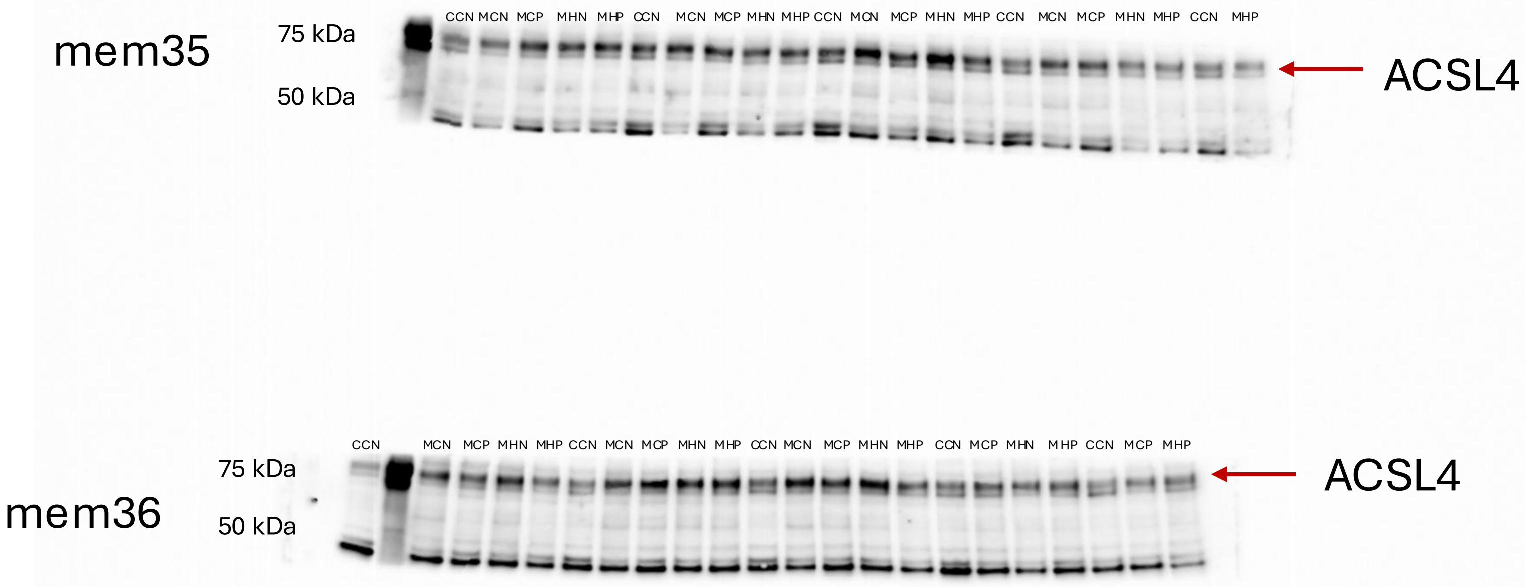
mem35
75 kDa
50 kDa
CCN MCN MCP MHN MHP CCN MCN MCP MHN MHP CCN MCN MCP MHN MHP CCN MCN MCP MHN MHP CCN MHP
ACSL4
mem36
75 kDa
50 kDa
CCN MCN MCP MHN MHP CCN MCN MCP MHN MHP CCN MCN MCP MHN MHP CCN MCP MHN MHP CCN MCP MHP
ACSL4

ALOX5

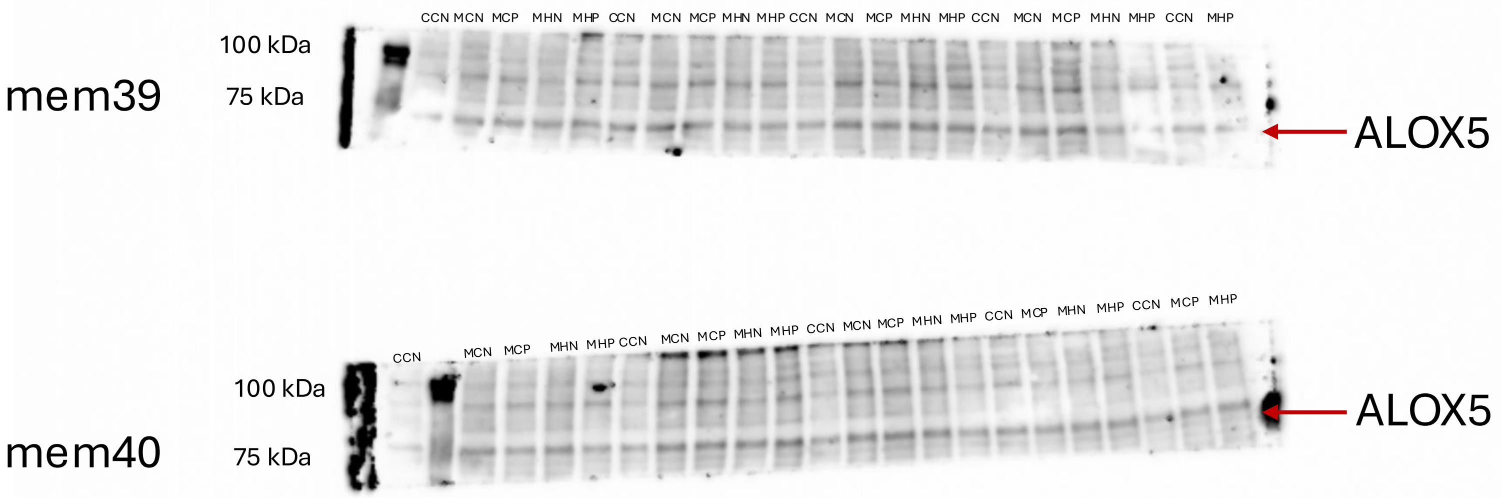
mem39
100 kDa
75 kDa
CCN MCN MCP MHN MHP CCN MCN MCP MHN MHP CCN MCN MCP MHN MHP CCN MCN MCP MHN MHP CCN MHP
ALOX5
mem40
100 kDa
75 kDa
CCN MCN MCP MHN MHP CCN MCN MCP MHN MHP CCN MCN MCP MHN MHP CCN MCP MHN MHP CCN MCP MHP
ALOX5

NOX4

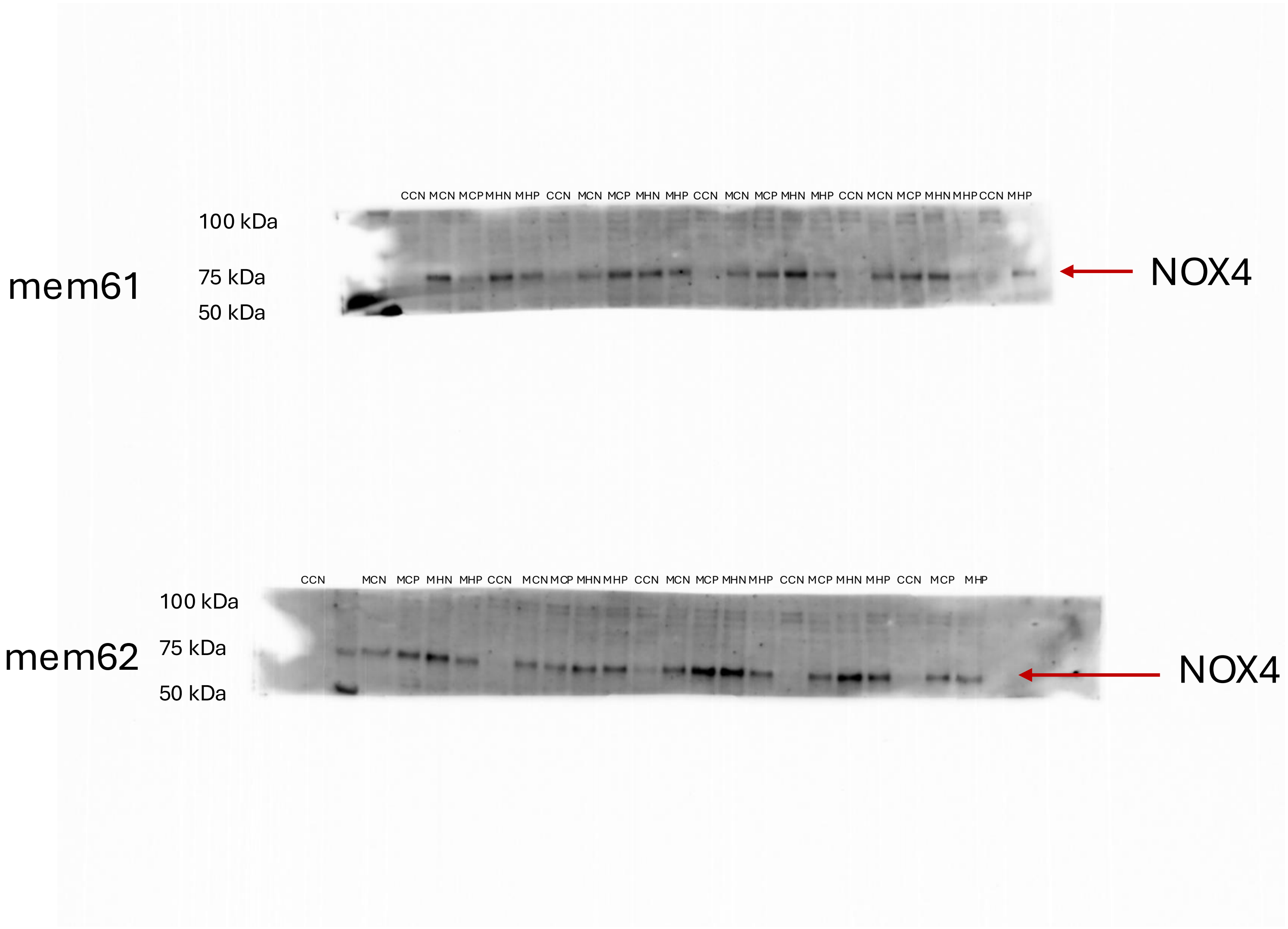

CCN MCN MCP MHN MHP CCN MCN MCP MHN MHP CCN MCN MCP MHN MHP CCN MCN MCP MHN MHP CCN MHP
100 kDa
mem61
75 kDa
50 kDa
NOX4
CCN MCN MCP MHN MHP CCN MCN MCP MHN MHP CCN MCN MCP MHN MHP CCN MCP MHN MHP CCN MCP MHP
100 kDa
mem62
75 kDa
50 kDa
NOX4

MDA

mem9

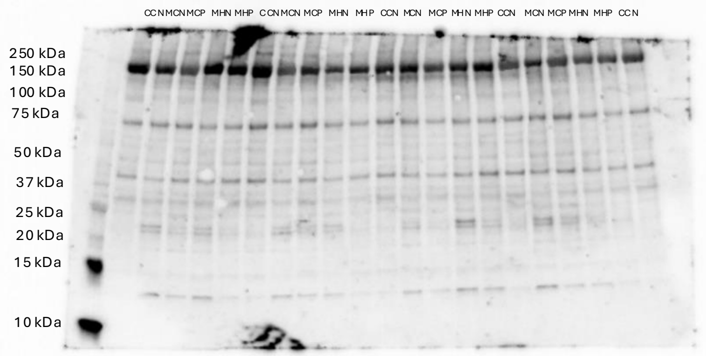

mem10

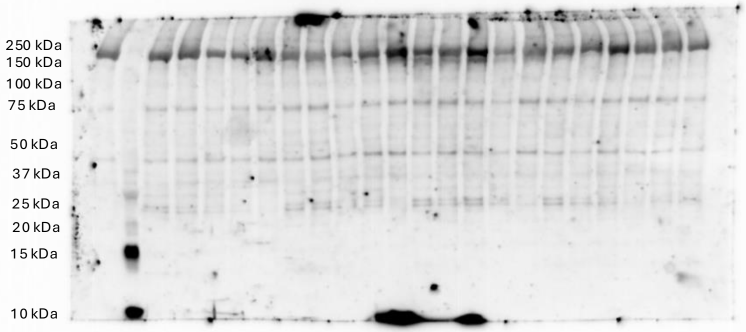

4HNE

mem15

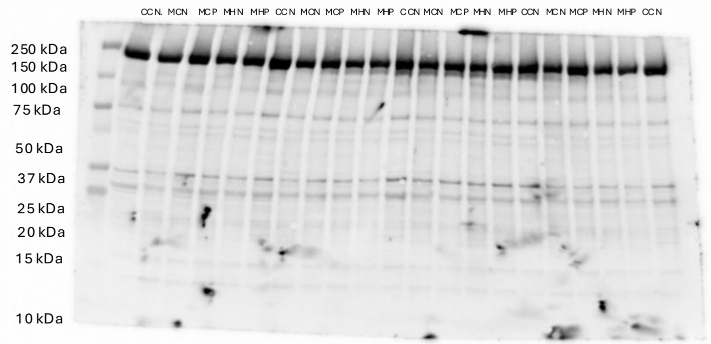

mem16

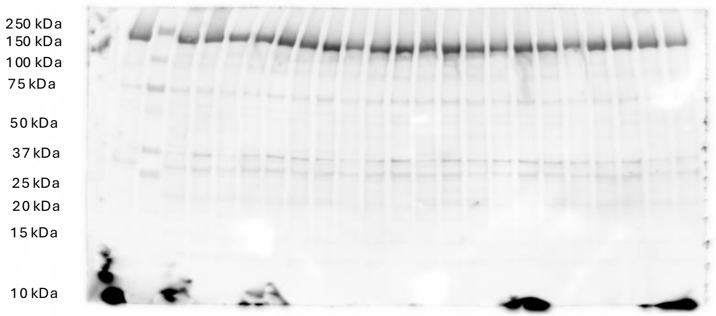

FSP1

mem59

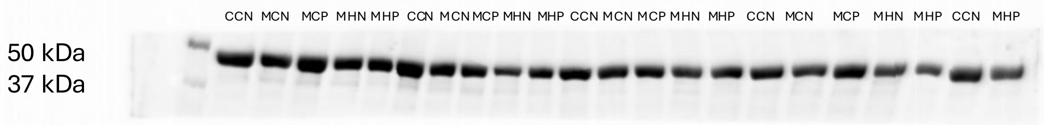

mem60

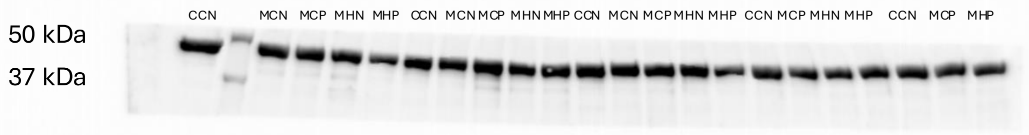

PRDX6

mem51

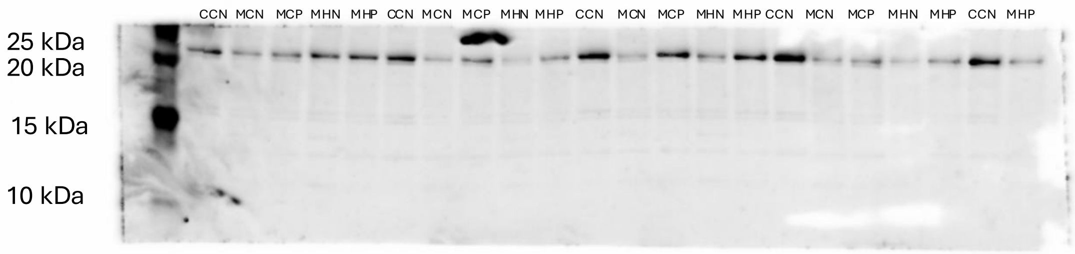

mem52

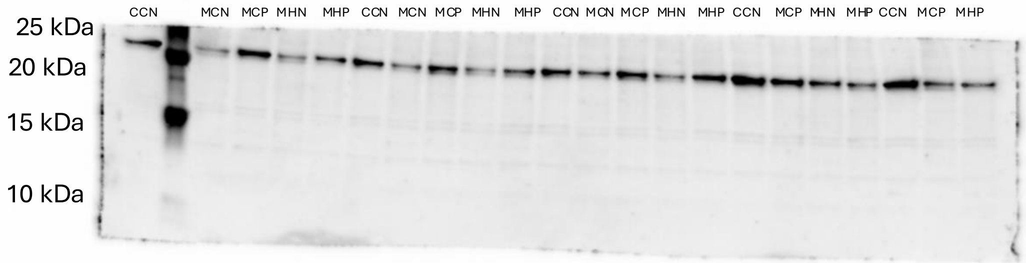

GPX4

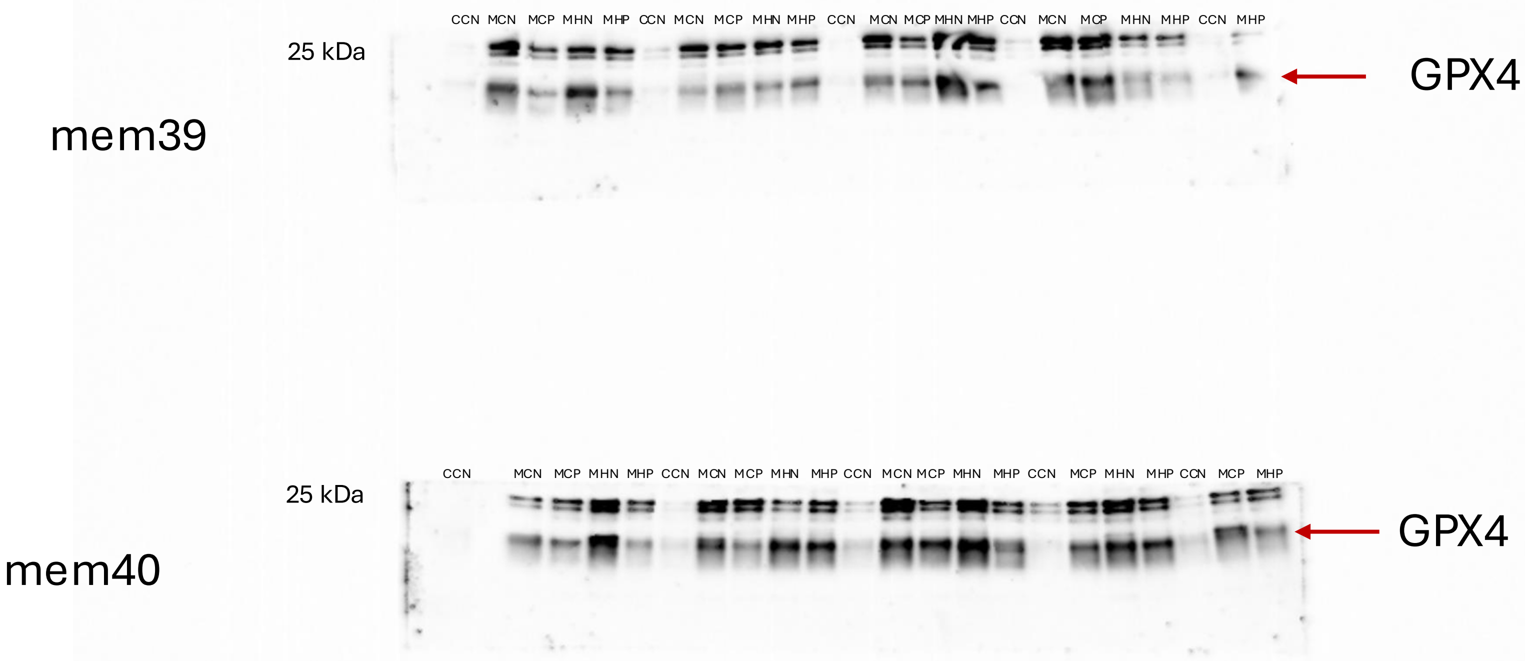

CCN MCN MCP MHN MHP CCN MCN MCP MHN MHP CCN MCN MCP MHN MHP CCN MCN MCP MHN MHP CCN MHP
25 kDa
mem39
GPX4
CCN MCN MCP MHN MHP CCN MCN MCP MHN MHP CCN MCN MCP MHN MHP CCN MCP MHN MHP CCN MCP MHP
25 kDa
mem40
GPX4

SOD1

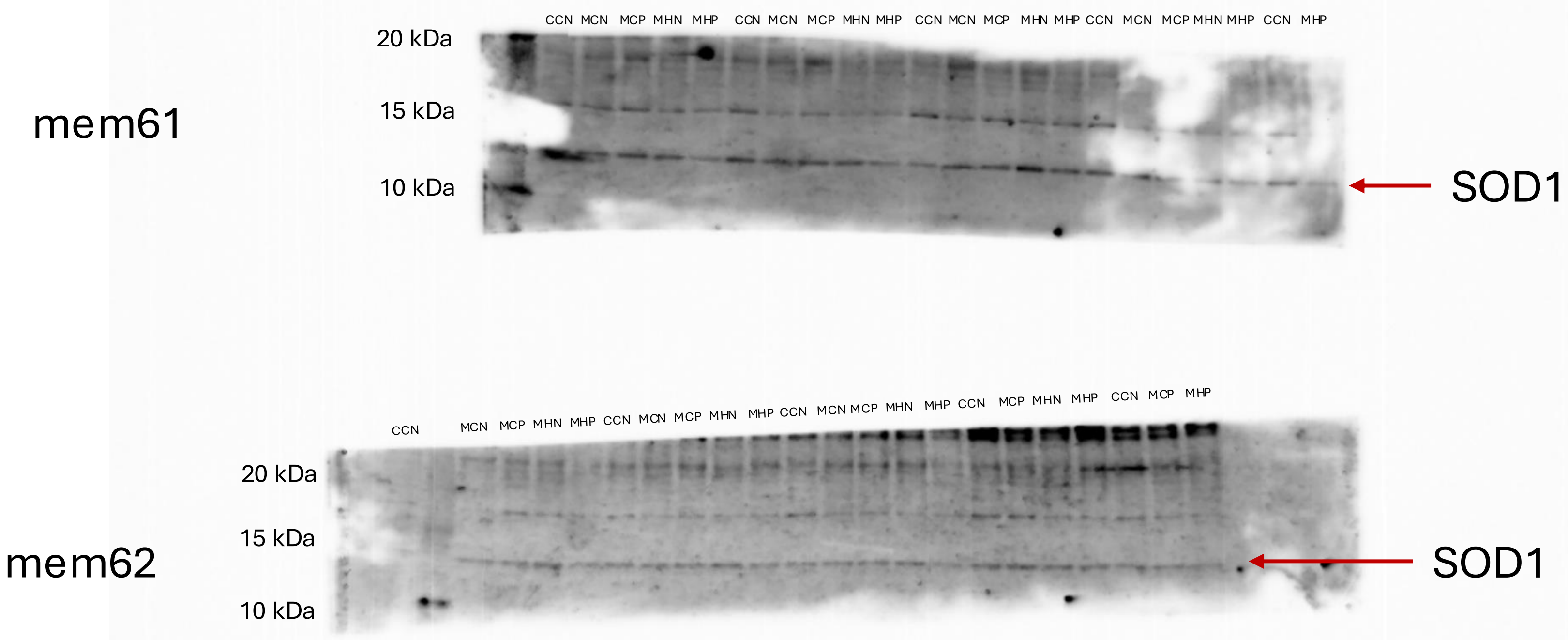
mem61
CCN MCN MCP MHN MHP CCN MCN MCP MHN MHP CCN MCN MCP MHN MHP CCN MCN MCP MHN MHP CCN MHP
20 kDa
15 kDa
10 kDa
SOD1
mem62
CCN MCN MCP MHN MHP CCN MCN MCP MHN MHP CCN MCN MCP MHN MHP CCN MCP MHN MHP CCN MCP MHP
20 kDa
15 kDa
10 kDa
SOD1

SOD2

mem61

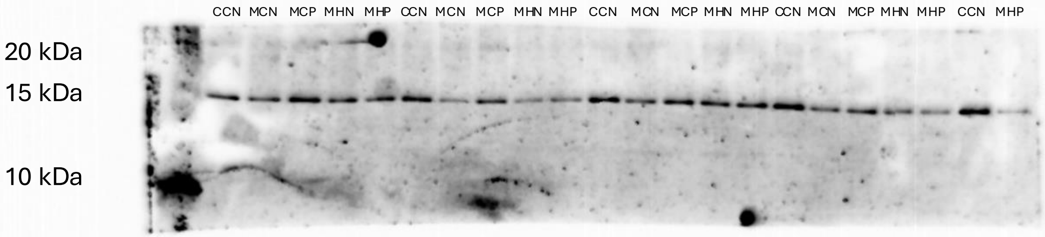

mem62

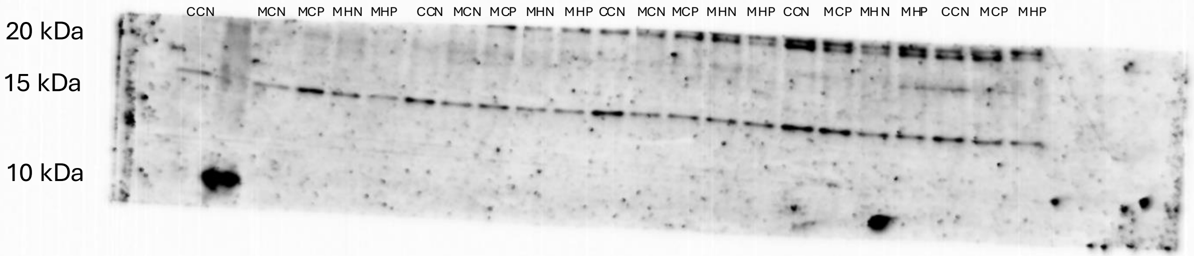

Catalase

mem61

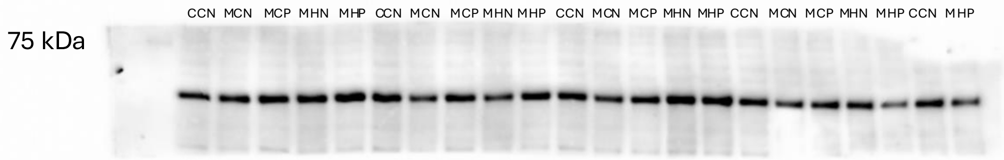

mem62

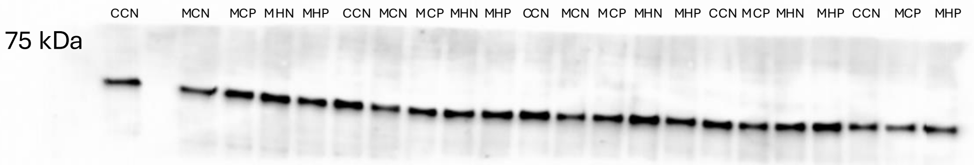

GPX1

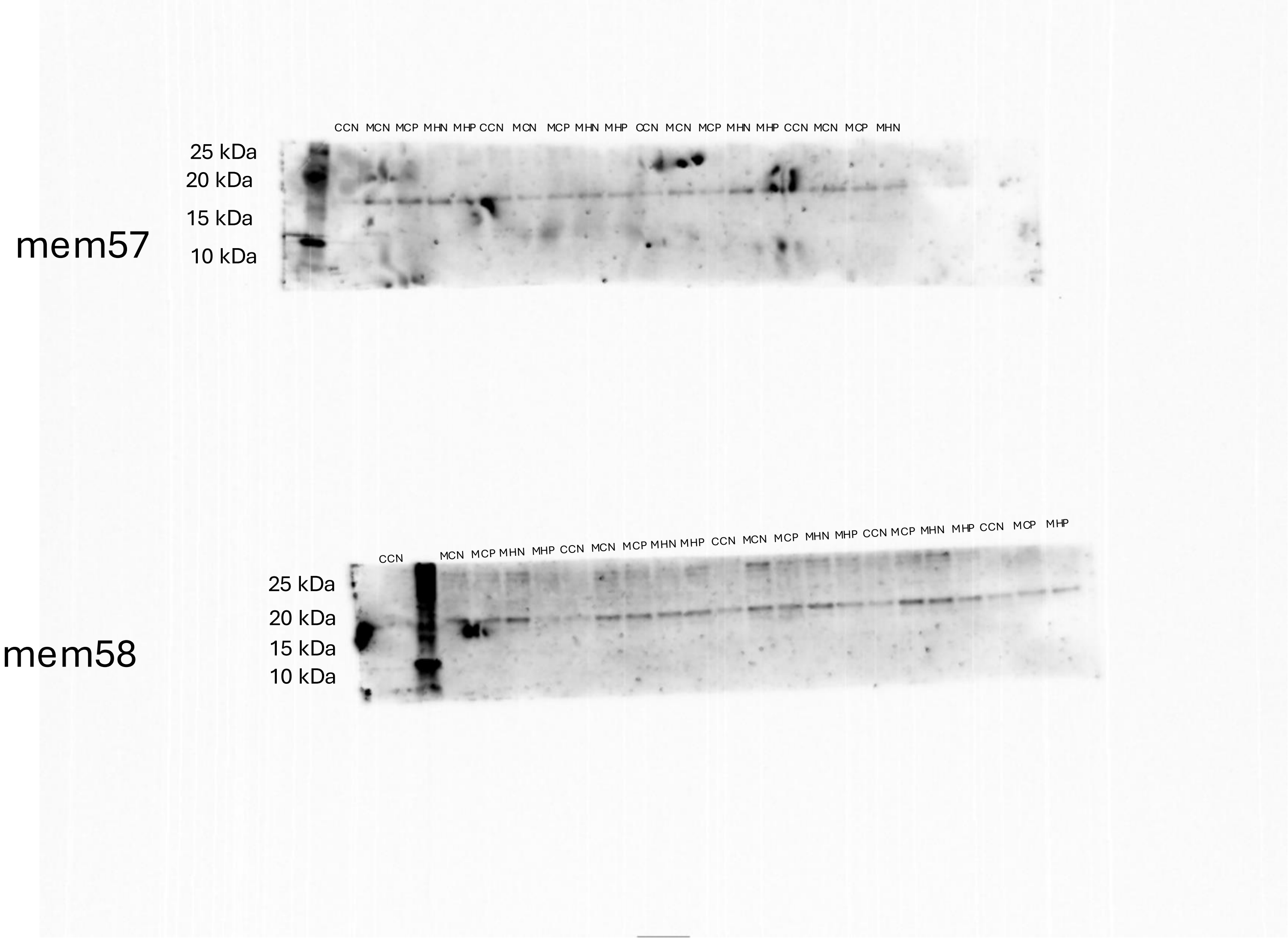
CCN MCN MCP MHN MHP CCN MCN MCP MHN MHP CCN MCN MCP MHN MHP CCN MCN MCP MHN
25 kDa
20 kDa
15 kDa
mem57
10 kDa
CCN MCN MCP MHN MHP CCN MCN MCP MHN MHP CCN MCN MCP MHN MHP CCN MCP MHN MHP CCN MCP MHP
25 kDa
20 kDa
mem58
15 kDa
10 kDa

Representative Ponceau S Stains

Figure S1

mem56

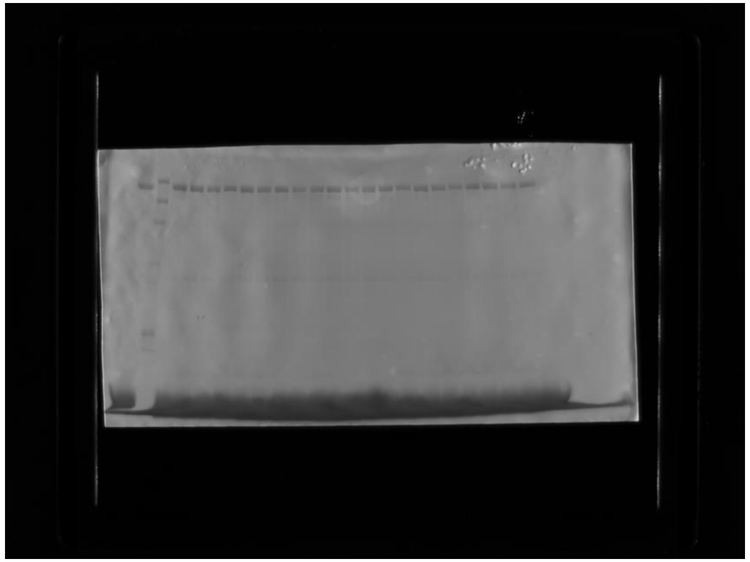

Figure S2

mem9

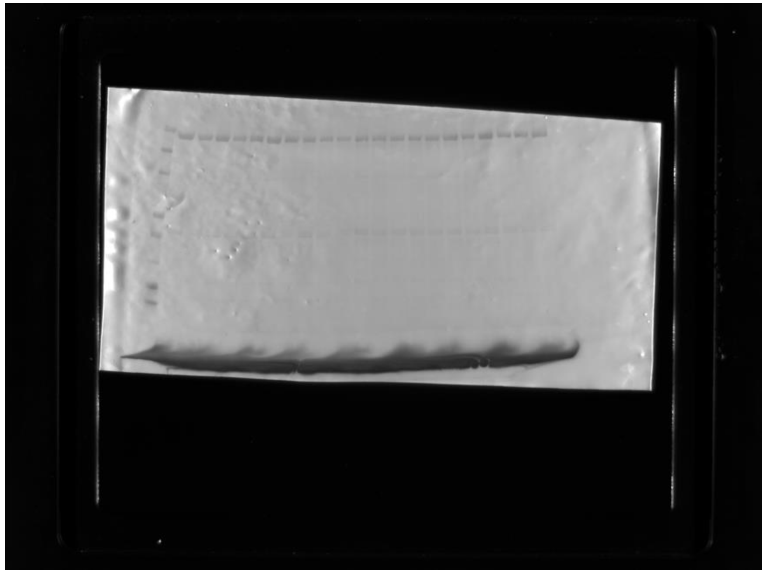

Figure S3

mem39

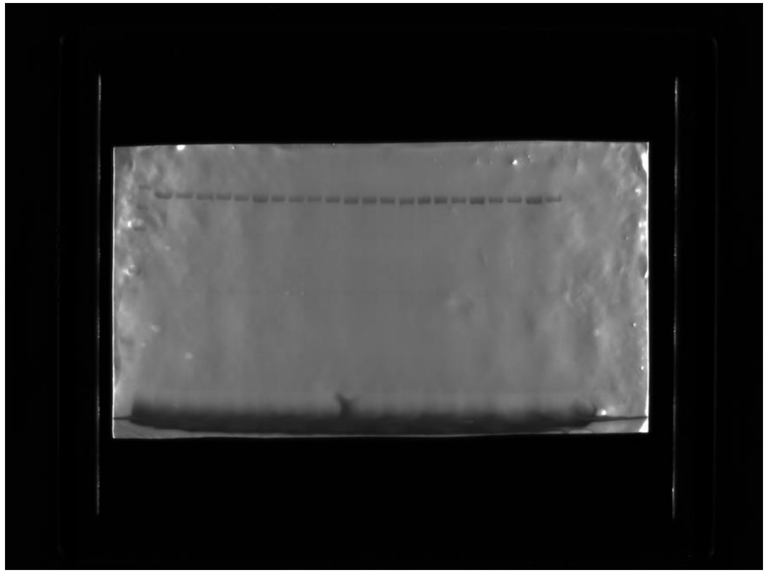

Supplement: Supplementary file 1 [file biomolecules-16-01347-s001.zip › biomolecules-4518036-supplementary.pdf]
